# Conformance of a 3T radiotherapy MRI scanner to the QIBA Diffusion Profile

**DOI:** 10.1002/mp.15645

**Published:** 2022-04-11

**Authors:** Madeline E. Carr, Kathryn E. Keenan, Robba Rai, Michael A. Boss, Peter Metcalfe, Amy Walker, Lois Holloway

**Affiliations:** ^1^ Centre for Medical and Radiation Physics University of Wollongong Wollongong Australia; ^2^ Medical Physics Ingham Institute for Applied Medical Research Liverpool Australia; ^3^ Department of Medical Physics Liverpool and Macarthur Cancer Therapy Centres Liverpool Australia; ^4^ Physical Measurement Laboratory National Institute of Standards and Technology Boulder USA; ^5^ Institute of Medical Physics University of Sydney Camperdown Australia; ^6^ Imaging Core Laboratory American College of Radiology Philadelphia USA; ^7^ South Western Sydney Clinical School University of New South Wales Liverpool Australia

**Keywords:** apparent diffusion coefficient, diffusion‐weighted imaging, quantitative imaging biomarker alliance, quantitative magnetic resonance imaging, reproducibility

## Abstract

**Purpose:**

To assess the technical performance of the apparent diffusion coefficient (ADC) on a dedicated 3T radiotherapy scanner, using a standardized phantom and sequences. Investigations into factors that could impact the technical performance of ADC in the clinic were also completed, including changing the slice‐encoded imaging direction and the reference sample ADC value.

**Methods:**

ADC acquisitions were performed monthly on an isotropic diffusion phantom over 1 year. Measurements of ADC %bias, coefficients of variation for short‐/long‐term repeatability and precision (CV_ST_/CV_LT_ and CV_P_), and *b*‐value dependency (Dep*_b_*) were calculated. The measurements were then assessed according to the Quantitative Imaging Biomarker Alliance (QIBA) Diffusion Profile specifications.

**Results:**

The average of all measurements over the year was within Profile recommended ranges. This included when testing was performed in different imaging directions, and on samples that had different ADC reference values (0.4–1.1 μm^2^/ms). Results in the axial plane for the central water vial included a bias of +0.05%, CV_ST_ /CV_LT_/CV_P_ = 0.1%/ 0.9%/0.4% and Dep*_b_* = 0.4%.

**Conclusions:**

The technical performance of ADC on a radiotherapy dedicated MRI scanner over the course of 12 months was considered conformant to the QIBA Profile. Quantifying these metrics and factors that may affect the performance is essential in progressing the use of ADC clinically: ensuring that the observed change of ADC in a tissue is due to a physiological response and not measurement variability.

## 1 INTRODUCTION

Quantitative Magnetic Resonance Imaging (qMRI) is increasingly used in radiation oncology. One technique involves Diffusion‐Weighted Imaging (DWI), which can be used to study a patient's tissue cellularity via the water Apparent Diffusion Coefficient (ADC). This has potential to aid in disease diagnosis and monitoring/predicting treatment responses.^1^, ^2^ However, the technical performance uncertainties associated with measuring ADC are currently limiting the widespread clinical implementation of this technique.^3^, ^4^, ^5^, ^6^

In 2019, the Quantitative Imaging Biomarker Alliance (QIBA) released the QIBA Diffusion Profile.^3^, ^7^ Specifically, the Profile lists precise methods and specifications that need to be met for a site to ensure reproducible ADC in multicenter trials. This incorporates recommendations based on literature to use standardized phantoms and sequences to establish a scanner's baseline performance levels (e.g., ADC %bias, repeatability, precision, signal‐to‐noise ratio [SNR]).^1^, ^4^, ^7^, ^8^ The Profile requires testing to be performed on a pure water ADC sample,^7^ and there are several phantoms available for this purpose.^6^, ^9^, ^10^, ^11^

The National Institute of Standards and Technology (NIST), National Cancer Institute (NCI), and Radiological Society of North America (RSNA) collaboratively developed a commercially available isotropic diffusion phantom, with 13 vials embedded with well‐characterized reference ADC values.^11^, ^12^ This phantom provides the ability to assess a scanner's technical performance over a wide range of physiologically relevant ADC values, which could affect ADC reliability.^11^, ^13^, ^14^ The NIST phantom has been used in the past to test the accuracy and reproducibility of ADC on/or between MRI scanners,^11^, ^15^, ^16^, ^17^ MR‐Linear accelerators,^5^, ^18^ and Diffusion Tensor Imaging (DTI) parameters on MRI scanners.^13^

The literature is however lacking long‐term and frequent system stability measurements, essential for simulating serial scanning in treatment response monitoring.^7^ Further, most studies investigating ADC reliability using the NIST phantom report only on using coronal^11^, ^15^, ^19^ or axial^5^, ^6^ slice‐encoded image acquisitions. Although the Profile requires only axial imaging for conformance testing, multidirectional DWI imaging is routinely performed in the clinic, depending on the anatomical site.^20^, ^21^, ^22^ Thus, it is important to determine any imaging directional dependencies on ADC reliability.^7^

The main aim of this study was to assess the long‐term technical performance of ADC on a 3T radiotherapy dedicated MRI scanner. Factors important for the clinical imaging of different anatomical sites with uncertain effects on scanner performance, including multidirectional imaging and ADC linearity, were also investigated.

## 2 MATERIALS AND METHODS

### 2.1 NIST diffusion phantom

**FIGURE 1:**
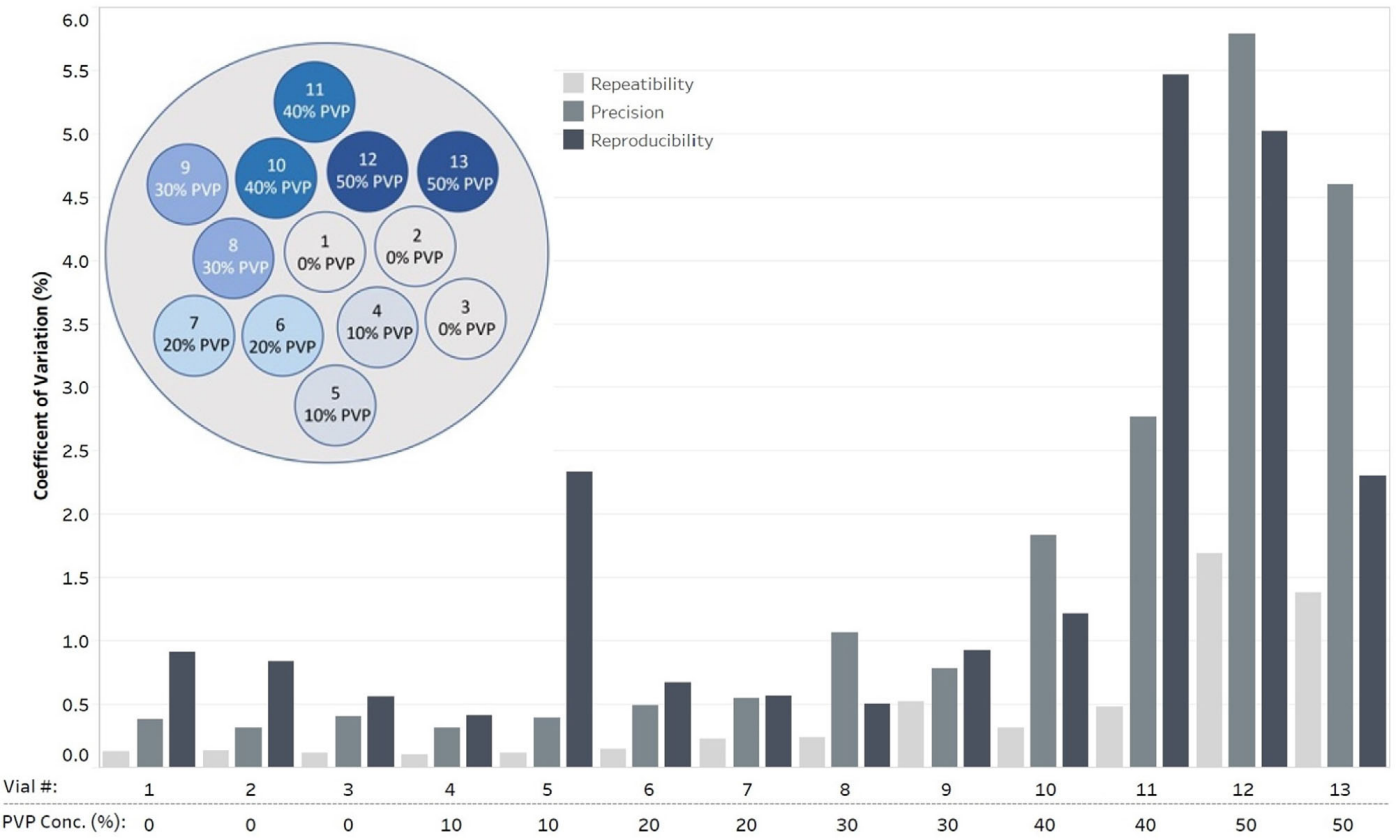
Repeatability coefficient of variation (CV_ST_), precision (CV_P_) and reproducibility (CV_LT_) derived for each vial for axial acquisitions, calculated as an average over the 12‐month study. A vial arrangement diagram has been included in the top left for positional reference for all 13 vials. PVP concentrations (conc.) are shown (by mass fraction (%)) for inner‐ and outer‐ring vials. Note that the central vial is to be positioned at isocenter and contains distilled water

The design of the diffusion phantom, manufactured by CaliberMRI (Colorado, USA), has been described in previous literature.^3^, ^11^, ^13^ Figure 1 shows the phantom's inner‐ and outer‐rings of vials surrounding the central distilled‐water vial. The surrounding vials contained a range of concentrations (by mass fraction) of polyvinylpyrrolidone (PVP) in aqueous solution including (%): 0, 10, 20, 30, 40, and 50. Reference ADC values of each of the vials (phantom serial#: DP128‐A‐03‐0113) are summarized in Table S1, covering a wide range of physiologically relevant ADC values.^14^

### 2.2 Image acquisition and phantom setup

The phantom was imaged at monthly intervals over 1 year (at a minimum of 2 weeks apart) using a 3T MRI scanner (Siemens Healthineers, MAGNETOM Skyra, Erlangen, Germany). The system‐specific phantom scan protocol and parameters used in this study are outlined in the QIBA Profile.^7^ This included the use of a 2‐D single shot echo‐planer imaging (SS‐EPI) sequence (scan time ≈ 2 min), with a 3‐scan trace and four *b*‐value weightings (s/mm^2^): 0, 500, 900, and 2000. The echo‐time (TE) and repetition‐time (TR) used for imaging were 10 000 ms and 106 ms, respectively.

Prior to scanning, the phantom was filled with an ice‐water bath and refrigerated for a minimum of 2 h to achieve thermal equilibrium at 0°C.^7^, ^14^ Immediately prior to scanning, the phantom was refilled with ice and the temperature was measured using a NIST‐traceable thermometer (Traceable® Extreme Accuracy Thermometer, 1227U09, Thomas Scientific, Swedesboro, USA). Temperature was measured again immediately after scanning.

For phantom alignment, the central water vial was aligned to isocenter within a 20 channel Head/Neck coil (Figure S1). The phantom was manually repositioned from its axial orientation to coronal and then sagittal within the Head/Neck coil (as described in the phantom manual).^14^ Simultaneously to each physical rotation, the slice‐encoding (and phase‐encoding for coronal) direction was changed to match the respective phantom orientation, maintaining Figure 1’s vial arrangement in the generated ADC maps. Each long‐term (LT) monthly acquisition included repeating the SS‐EPI sequence four times to acquire the short‐term (ST) measurements in each phantom orientation, as per QIBA Profile guidelines.

Both trace‐DWI and scanner‐generated (inline) ADC maps were exported from the Siemens Syngo Workstation to preserve DICOM metadata. The inline maps were calculated using a linear regression analysis by fitting the signal for all *b‐*values, S(b),to the monoexponential model (Equation 1).^23^

(1) $$S\left(b\right)=S_{0}e^{-b\cdot \mathrm{ADC}}$$

Note$\;S_{0}$ denotes the signal intensity when the *b*‐value = 0 s/mm^2^.

### 2.3 Region of interest analysis

Using the first repetition ADC map measured for each imaging direction, the central pixel location was manually identified for each of the vials using ImageJ v1.53c (National Institutes of Health, Maryland, USA). These locations along with all four repetitions of trace‐DWI and ADC maps were imported into an in‐house developed Python analysis script. Circular regions of interest (ROIs) of 1.2 cm diameter, covering approximately 109 pixels, were positioned over the center of each of the 13 vials on three central phantom slices for statistical analysis (average pixel intensity calculated over the volume of interest [VOI]). Phantom slices found to have major artifacts occurring near any of the 13 vials were excluded from analysis via shifting the entire VOI selection.

### 2.4 QIBA Profile analysis

The Profile required the assessment of seven key measurements, with calculations and definitions outlined in Table 1 and tolerance limits in Table 2. Measures for tests A–E were calculated using the inline derived ADC maps, while tests F and G required trace‐DWI images. Further details on the methods used to complete this testing can be found in the Profile documentation.^7^

**TABLE 1:** Overview of tests completed to assess conformance to the Profile^7^

**Test**	**Relevant Equations**	**Definitions**	
**A**	$\%\mathrm{bias}\;=\;\left(\frac{\mu \;-{\mathrm{DC}}_{T}}{{\mathrm{DC}}_{T}}\right)\times \;100\%$	DC_T_	= True diffusion coefficient
		μ	= Mean of measurements
**B/C**	$RC_{\mathrm{ST}/\mathrm{LT}}=\;2.77\;\times \mathrm{SD}$	RC	= Repeatability coefficient
	$CV_{\mathrm{ST}/\mathrm{LT}}=\;100\%\;\times \;\frac{\mathrm{SD}}{\mu \;}$	CV	= Coefficient of variation
		SD	= Standard deviation
		_ST_	= Over 4 × short‐term measurements
		_LT_	= Over 12 × long‐term measurements
**D**	$R^{2}=\;1-\;\frac{\mathrm{RSS}}{\mathrm{TSS}}$ AND $Y\;=\;\beta_{0}+\beta_{1}\times DC_{T}$	*R* ^2^	= Coefficient of determination
		RSS	= Sum of squares of residuals
		TSS	= Total Sum of Squares
		Y	= Measured ADC (all vials/months)
		β_0_	= Intercept
		β_1_	= Slope
**E**	$CV_{P}=\;100\%\;\times \;\frac{SD_{\mathrm{pix}}}{\mu_{\mathrm{ROI}}\;}$	ROI	= Region of interest (isocenter vial)
		$SD_{pix}$	= Over ADC values within the ROI
**F**	$\mathrm{SNR}\;=\;\frac{\mu_{\mathrm{ROI}\;}\left[\mathrm{Signal}\;\mathrm{image}\right]}{\mu_{\mathrm{ROI}\;}\left[\mathrm{Noise}\;\mathrm{image}\right]}$	SNR	= Signal to noise ratio
		Signal	= Average of pixel values for each ROI over the 4 x ST repetitions
		Noise	= Average of pixel SD values for each ROI over the 4 x ST repetitions
**G**	$\mathrm{De}p_{b}=\;100\%\;\times \left|\frac{\mathrm{AD}C_{b0,bn+1}-\mathrm{AD}C_{b0,bn}\;}{\mathrm{AD}C_{b0,bn}}\right|$	Dep_*b*_	= *b*‐Value dependence
		$\mathrm{AD}C_{b0,bn+1}$	= ADC generated using *b*_0 _= 0 s/mm^2^ and *b_n_*_+1_, where *b_n_*_+1 _> *b_n_*
		$b_{1-3}$	= 500, 900, or 2000 s/mm^2^

*Note*: Excluding test D, Profile testing was only required to be performed on the central water vial (at isocenter) using axial acquisitions. Further, short‐term (ST) refers to the intraday measurements acquired, while long‐term (LT) refers to the intramonth measurements acquired

**TABLE 2:** Accuracy, repeatability, reproducibility, linearity, random error, SNR, and *b*‐value dependence (tests A →G) tolerance limits and mean value ± SD (where applicable), obtained from the 12 monthly measurements of the central water vial (as per Profile requirements)

**Test**	**Performance metric**	**Profile tolerance**	**Axial result**	**Coronal result**	**Sagittal result**
**A**	|bias (%)|	≤ 3.60	+0.05 ± 0.01	+0.83 ± 0.00	+0.29 ± 0.01
**B**	RC_ST_ (μm^2^/ms)	≤ 0.015	0.003 ± 0.001	0.005 ± 0.002	0.003 ± 0.001
	$CV_{\mathrm{ST}}\;\left(\%\right)$	≤0.5	0.1 ± 0.0	0.1 ± 0.1	0.1 ± 0.0
**C**	RC_LT_ (μm^2^/ms)	≤0.065	0.028	0.011	0.027
	$CV_{\mathrm{LT}}\;\left(\%\right)$	≤ 2.2	0.9	0.3	0.9
**D**	*R* ^2^	> 0.9	1.0	1.0	1.0
	$\mathrm{Slope}\;(\beta_{1})$	0.95 ≤$\;\beta_{1}\;$≤ 1.05	1.00	1.02	1.02
**E**	CV_P_ (%)	<2	0.38 ± 0.10	0.43 ± 0.04	0.38 ± 0.04
**F**	SNR^a^	≥ 50 ± 5	332 ± 146	269 ± 93	356 ± 68
**G**	Dep_*b*_(%)^a^	<2	0.4 ± 0.3	0.3 ± 0.2	1.4 ± 2.1

^a^Certain month's data have been excluded from the presented results due to retrospective findings of signal saturation occurring within the data sets. For SNR, this included excluding axial results acquired for months 1, 2, 6, and 9, and coronal and sagittal results for months 1 and 5, and 1 and 6, respectively. For Dep*_b_*, results for month 1 were excluded in calculations for all directions

### 2.5 Software validation and spatial dependence

The QIBA Profile recommended investigating the analysis software used for testing Profile conformance. To do this, computer‐generated DICOM data sets known as digital reference objects (DROs), with *b*‐values of 0, 500, 800, and 2000 s/mm^2^ were imported into the offline DWI‐fit Python script.^24^ The offline fitting method was alike that described for the inline ADC map derivation.

Offline ADC maps of the DROs were produced to estimate the %bias and standard deviation (SD) over a range of phantom relevant SNRs (50–100) and ADC values (0.1–1.1 μm^2^/ms).^7^, ^25^ The same fit was used on the first repetition of each monthly trace‐DWI from the axial phantom scans (data sets = 12), using the central water vial ROI from tests A–E. *R* ^2^ was used as a measure of goodness‐of‐fit in both cases, and inline versus offline ADC values were compared to assess %bias. The offline script was also designed to identify ROIs in the trace‐DWI images that had experienced signal saturation. Signal saturation, also known as data clipping, occurs when the signal received is outside of the system's detectable range. In addition, the same SNR code as used in test F was used on the four repetition DROs available.^24^

Following personal communication with QIBA, an estimate of spatial dependence (Dep*_S_*) was completed using the diffusion phantom. Specifically, the %bias deviations along the lengths of the central water vial (axially), outer‐ring water vial (axially), and central water vial (coronally) were respectively used to assess superior to inferior (SI), right to left (RL), and anterior to posterior (AP) spatial dependencies at approximately 4 cm from isocenter. A Profile tolerance of ±4% was stipulated for each individual direction.^7^

## 3 RESULTS

The phantom was imaged 12 times over a 1‐year period, with an average four‐week interval between imaging sessions. Average pre‐ and postscan temperatures were –0.1 ± 0.1°C and 0.0 ± 0.2°C, respectively. For any monthly imaging session, the maximum (absolute) temperature changes pre‐ and postscanning was 0.4°C.

Typical DWI‐trace and inline ADC maps (and respective ROIs) are shown in Figure S2. Susceptibility‐induced distortions in the ADC maps were primarily observed in outer ring 40% and 30% PVP vials for axial and sagittal acquisitions, respectively (Figure S3). Consequently, central VOIs were selected to mitigate the observed distortions. A summary of the Profile test tolerances and acquired results for the central water vial are listed in Table 2.

### 3.1 ADC accuracy (A), repeatability(B), reproducibility (C), linearity (D), and precision (E)

Figure 1 highlights that Profile tolerance limits for repeatability, reproducibility (excluding the axial CV_LT_ of the outer‐ring 10% PVP vial), and precision were met for all vials (1–8) with concentrations 0–30% PVP (ADC range: 0.4–1.1 μm^2^/ms). The average ADC calculated for all directions/vials can be found in Table S1 and Figure S4. In general, vials with lower diffusivities (higher concentrations of PVP) had inferior performance metrics, and even the sign of the %bias measurement varied for different imaging directions. The inner‐ring 50% PVP vial (Figure 1) had an axial bias up to +13.61% and CV_LT_ = 5.8%, and sagittal bias of –21.82% and CV_LT_ = 7.4%.

**FIGURE 2:**
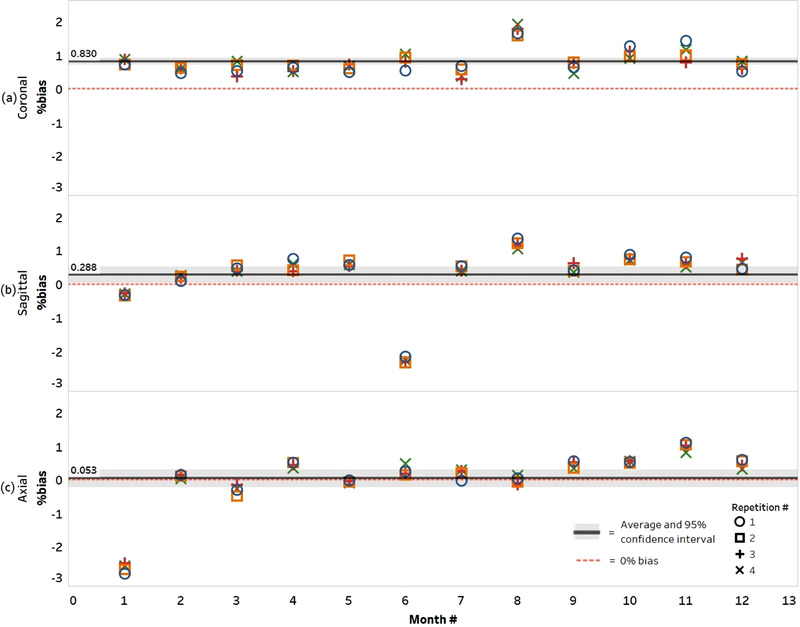
Bland–Altman plots for the central water vial (reference ADC = 1.109 μm^2^/ms) coronal (a), sagittal (b), and axial (c) acquisitions show the difference (%bias) between measured and reference ADC values over the 12‐month study. The average %bias (and 95% confidence intervals) is displayed and includes +0.830% (+0.724 to +0.936), +0.288% (+0.042 to +0.534), and +0.053% (–0.204 to +0.309) for coronal, sagittal, and axial acquisitions, respectively

An overview of monthly %bias results for the central water vial are presented in Figure 2 for all repetitions and each imaging direction. It can be observed that the %bias was well within Profile tolerance range (±3.60%). From this figure, it is also evident that all within‐session repetitions generated similar magnitude ADC values, whereas monthly repetitions fluctuated (around 0% bias). Specifically, no monotonic trends in ADC variability with time were found over the four within‐session repetitions, nor were any changes in artifacts observed.

**FIGURE 3:**
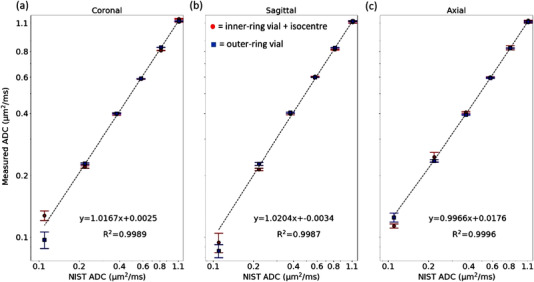
Correlation plots for coronal (a), sagittal (b), and axial (c) acquisitions with measured ADC values compared to the NIST reference ADC values for each vial. Note that all axes have employed a logarithmic scale and error bars are given as the standard deviation found between all 12 months ADC values

Using all 13 vials average ADC over the duration of the 12‐month study, a strong, positive, and linear correlation was found (Figure 3) between measured and reference ADC values for all directional acquisitions (*R* ^2 ^> 0.99). Similarly, the slopes (β_1_) in Figure 3 were all within the Profile tolerance range. It can be observed that all inner‐ and outer‐ring vials of the phantom performed similarly, excluding 40% and 50% PVP vials (lowest ADC), which also had the largest SDs.

### 3.2 SNR (F) and software dependence

On the axial *b*‐value = 0 s/mm^2^ magnitude images, pixel signal intensities in the ROI of the central water vial saturated for months 1, 2, 6, and 9. This resulted in failed SNR calculations (high signal and minimal noise), and thus the SNR presented in Table 2 is an average over only 8 months of repetitions. Similarly, the coronal and sagittal acquisitions experienced saturation in the central water vial for months 1 and 5 and months 1 and 6, respectively. Thus, the SNR was only calculated over 10 months in both cases.

In assessing the image analysis software, equivalence (within SD) of inline versus offline axial central water vial ADC values were found: 1.110 ± 0.010 and 1.112 ± 0.010 μm^2^/ms, respectively. A strong correlation (*R* ^2^ > 0.993) between the two methods’ measured ADC values were found when including all vials, with the inline method slightly underestimating the ADC on average by 0.2%. The offline derived ADC value fluctuations is shown in Figure S5, highlighting signal saturation minimally affected the ADC (for months 2, 6, and 9). However, larger SDs were found for saturated fits, especially for month 1.

For the DRO study, a goodness‐of‐fit of *R* ^2 ^= 0.995 was found with %bias ± SD remaining within the ≤3.60% tolerance for most ADC/SNR combinations in phantom relevant ranges (Figure S6). For the DRO ROI of ADC = 1.1 μm^2^/ms and SNR = 100, a slight overestimation of SNR was found, 104.9 ± 4.5 (with 95% confidence interval [CI]), using the SNR analysis method implemented for test F.

### 3.3 *b*‐Value (G) and spatial dependence

Note that month 1 results were excluded from this test due to considerable signal saturation effects causing axial *b*‐value dependencies to be up to a maximum of 11.8% for pairs *ADC*_0,500_ and *ADC*_0,900_. When considering only the remainder 11 months of measurements, axial *b*‐value dependence was largest when calculated for the *ADC*_0,500_ and *ADC*_0,2000_ pair: 0.6 ± 0.4% (mean ± SD). This was followed by respective dependencies of 0.4 ± 0.2% for pairs *ADC*_0,500_ and *ADC*_0,900_ and 0.3 ± 0.1% for pairs *ADC*_0,900_ and *ADC*_0,2000_. Further considering the later 11 months for both axial and coronal plane measurements, all pair dependencies stayed below 1.3% and were within a 0.4% difference from the monthly average of all pairs. Sagittal *b*‐value dependence, however, performed outside of Profile tolerance limits. This was largely due to month 6's sagittal measurements (affected by saturation), causing high dependencies of up to 12.0% for pairs *ADC*_0,500_ and *ADC*_0,2000_.

For spatial dependence, AP bias on average over all months was +0.71 ± 0.85%, followed by SI at ±0.43 ± 1.60% and RL of +0.34 ± 1.45%. All %bias stayed well within the ±4% tolerance on average; however, SI varied substantially for months 1 and 2.

## 4 DISCUSSION

In this study, the ADC derived on a 3T dedicated radiotherapy MRI scanner was found to be accurate, repeatable, and reproducible using systematic image acquisitions over 1 year. By using a standardized set of testing procedures such as the QIBA Profile, the ADC measured by the scanner in prospective single or QIBA‐certified multisite patient‐based studies can be said to be reliable, with negligible contributions to ADC due to systematic errors.^6^, ^7^

The average axial %bias (+0.05%) and repeatability (CV_ST_ = 0.1%) for the central water vial (at isocenter) were measured to be well within conformance limits and were comparable to measurements reported in the literature: %bias < ±4.3% and CV_ST _< 3.2%.^5^, ^6^, ^11^, ^18^ Long‐term system stability of deriving the ADC (axial CV_LT _= 0.9%) was also found in this study. All acquisition parameters remained constant throughout the study, and the PVP solutions embedded in the phantom are known to be chemically stable.^10^ Previous studies using this phantom found similar results, with the CV_LT_ to be within ranges of <2.2% when acquiring 2 scans within 6 months on the same scanner,^18^, ^19^ and CV = 2.1% when comparing between multiple MRI scanners.^13^

Overall, this study showed there was minimal imaging directional dependence on ADC performance—a factor not investigated in previous studies using the same phantom. The worst accuracy/reproducibility was observed for vials with lower diffusivities (40% and 50% PVP), in agreement with the literature.^4^, ^11^, ^13^, ^17^, ^18^ Further in accordance with the literature,^11^, ^13^ there were no significant differences (*p* > 0.05) between inner‐ and outer‐ring vial ADC values measured.

The large deviations observed for the lower diffusivity vials could be due to several factors including eddy currents^7^; increased likelihood of susceptibility‐induced distortions occurring near the higher concentrated PVP vials; gradient nonlinearities, which can impact the ADC measured at farther distances from isocenter^18^; or from insufficient contrast to noise ratio (CNR) or SNR to correctly assess the ADC in the highly concentrated vials.^11^ It is important to note that the 40% and 50% PVP vials at 0°C have ADC values below physiological range.^17^

The signal saturation observed in this study has not been reported in patient‐based imaging within the department, nor in previous literature investigating ADC variability. This effect is not easy to detect: in the offline computed ADC maps, saturation was found to primarily affect the SDs derived from the DWI‐ADC fit, while minimally impacting the actual ADC value. Saturation was mainly investigated due to past studies completed on the same MRI scanner having identified similar effects when undertaking phantom imaging.^26^ Further investigations, out of the scope of this study, would be required to find the cause and factors affecting the signal saturation.

The *b*‐value is an important factor when considering patient image protocol optimizations. Given the ideal *b*‐value selection is dependent on properties of the anatomy, there is no simple way to determine the exact *b*‐value combinations that should be used.^5^, ^7^ Instead, there is often a compromise made between maintaining adequate SNR, while minimizing perfusion attributes to the acquired signal.^7^ *b*‐value combination recommendations for some anatomies, including for brain, liver, prostate, and breast, is provided in the Profile.

The *b*‐value dependency in this study for all *b*‐value pairs were within the 2% Profile tolerance (even when including the saturated data in month 1). Pair ADC_0,500_ and ADC_0,2000_ had the largest *b*‐value dependence. However, there was no significant difference (*p* > 0.05) between this pair and other combinations, including when all four *b*‐values were used for the offline fit. Since the reference sample tested was distilled water (known to demonstrate a monoexponential behavior), this finding was expected and in agreement with the literature.^6^, ^9^, ^18^

Adequate ADC fits and SNR calculations were found in this study by using offline analysis methods to analyze the DRO data sets. With only slight differences between inline and offline derived ADC values (correlation *R* ^2^ > 0.993), confidence was assured in utilizing the inline generated ADC maps for majority of analysis as per departmental request, to use the same analysis method as implemented for patient images acquired on the same scanner. The underestimation of inline derived ADC values compared to offline has been noted to occur in past studies and is likely vendor‐specific.^15^ Offline methods would need to be used for future multisite studies to ensure the occurrence of a standardized analysis pathway.^15^

The potential of temperature changes affecting the measured ADC, which can be up to 2.4%/°C,^1^, ^3^, ^6^ was removed using an ice‐bath. However, preparing the ice‐bath requires considerable time and can increase the occurrence of susceptibility‐induced distortions (which are known to commonly occur in EPI‐DWI acquisitions).^10^, ^17^ Quantifying the impact of these distortions on the ADC measurement would require further investigation.^27^ With the recent release of room‐temperature diffusion phantom reference ADC values, the need for ice‐baths should be minimized in future studies.^14^

For spatial dependency, the average %bias over the 12‐month study measured in each direction from isocenter was well within ±4% tolerance. It should be noted that the spatial offsets examined for this test were less than the offsets recommended by the Profile (±10 cm from isocenter). Consequentially, complete characterization of the spatial dependence could not be achieved. Recommendations for future investigations would include the use of a large homogeneous phantom for this assessment. Although this testing is less important for small fields of view, diffusion studies completed on relatively uniform anatomies like brain have been shown to be significantly impacted by effects such as gradient nonlinearities and thus should be monitored.^28^

Additionally, patient ADC values can also be biased by inadequate system SNR.^6^, ^7^ In this study, all directional SNRs were found to be sufficient and considerably higher than that found in past studies, which failed to meet conformance (however, such studies used 1.5 T MRI‐based systems).^6^, ^18^ It should be recognized that the results obtained in this study were for the assessment of baseline scanner performance. Given that phantoms lack tissue complexity, results presented in this study such as the SNR are likely superior when compared with patient‐based imaging.^29^ For assessing clinical conformance to anatomy‐specific Profile claims, in vivo test–retest assessments should be completed (e.g., for brain and prostate).^7^

There were other limitations in this study, including that imaging did not occur on days directly surrounding two scanner upgrades involving the replacement of the scanner's Transmit‐Box. Although no clear relationship between ADC value fluctuations and the timing of the upgrades were found, similar upgrades have been found to affect patient‐based ADC values in the past and should be closely monitored.^30^ Future investigations will involve completing similar baseline testing (although at less frequent intervals) in a multisite trial to validate results found, including changing the imaging direction and reference ADC values.

Findings from this study have led to department recommendations to conduct ADC QA testing annually, and directly before and after commencing a multicenter trial. This QA is in addition to performing the testing at times surrounding any major scanner upgrades. Specifically, completing this testing in only one imaging direction (axial as per QIBA guidelines) and on a pure water sample was considered sufficient following baseline performance measurements.

This study extends knowledge in understanding ADC long‐term variability on clinical MRI scanners. To the best of the authors’ knowledge, no prior study has reported in detail testing all aspects of the QIBA Diffusion Profile. Particularly, this study demonstrated Profile conformance over a wide range of physiological relevant ADC values and over three orthogonal imaging directions using a novel diffusion phantom. These are important findings for future clinical applications whereby patient and consequently phantom QA imaging is required in alternate directions and over different ADC valued anatomies.^1^, ^20^, ^21^, ^22^ Finding high reliability in the ADC values derived promotes the use of ADC in clinical trials to monitor and assess long‐term treatment responses, essential for progressing the clinical implementation of qMRI technology.

## 5 CONCLUSION

In this study, the technical performance of a 3T dedicated radiotherapy MRI scanner was quantified over a 12‐month period. Specifically, QIBA Profile conformance specifications were met, including adequate axial imaging accuracy (bias = +0.05%), repeatability (CV_ST _= 0.1%) and long‐term reproducibility (CV_LT _= 0.9%). While phantom‐based results can be effectively used to assess baseline scanner performance, test–retest patient‐based studies would be required to examine clinical conformance to anatomy‐specific Profile claims. Recommendations to the department regarding future ADC QA included completing conformance testing annually. This involves only axial imaging on a highly purified water sample: independent of the anatomical sites planned for prospective imaging.

## 6 DISCLAIMER

Certain commercial equipment, instruments, or materials are identified in this paper to foster understanding. Such identification does not imply recommendation or endorsement by the National Institute of Standards and Technology, nor does it imply that the materials or equipment identified are necessarily the best available for the purpose.

## CONFLICT OF INTEREST

Liverpool and Macarthur Cancer Therapy Centres have a master research agreement with Siemens. However, this work is independent of that agreement.

## Supporting information

Figure S1

Figure S2

Figure S3

Figure S4

Figure S5

Figure S6

Table S1

## Data Availability

Authors will share data upon reasonable request to the corresponding author.
