# Supplementary material for: Conformance of a 3T radiotherapy MRI scanner to the QIBA Diffusion Profile
Source: Med Phys. 2022 Apr 11;49(7):4508–17. doi: 10.1002/mp.15645 (PMC9543906; doi:10.1002/mp.15645)
Supplement: Supplementary file 1 — Figure S1 [file MP-49-4508-s004.pdf]

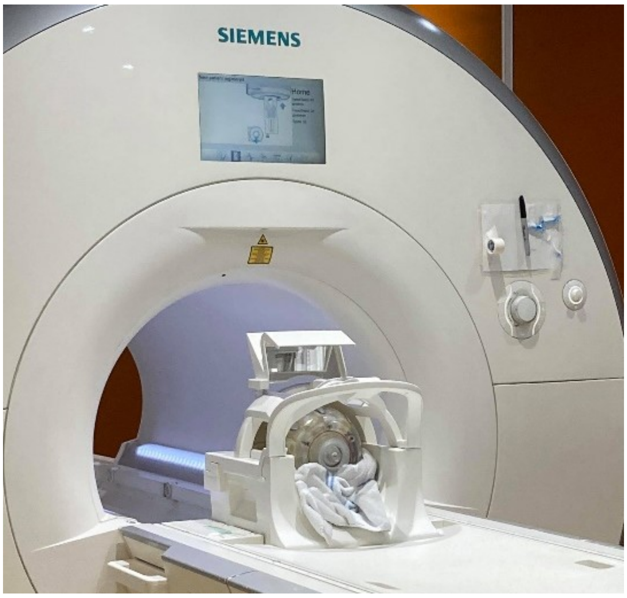

Supplementary Figure S-1: Diffusion phantom in its axial orientation within a head/neck coil. A 3D-printed base holder and stabilizing aids were used to assist in reproducible positioning.
