# Supplementary material for: Conformance of a 3T radiotherapy MRI scanner to the QIBA Diffusion Profile
Source: Med Phys. 2022 Apr 11;49(7):4508–17. doi: 10.1002/mp.15645 (PMC9543906; doi:10.1002/mp.15645)
Supplement: Supplementary file 2 — Figure S2 [file MP-49-4508-s003.pdf]

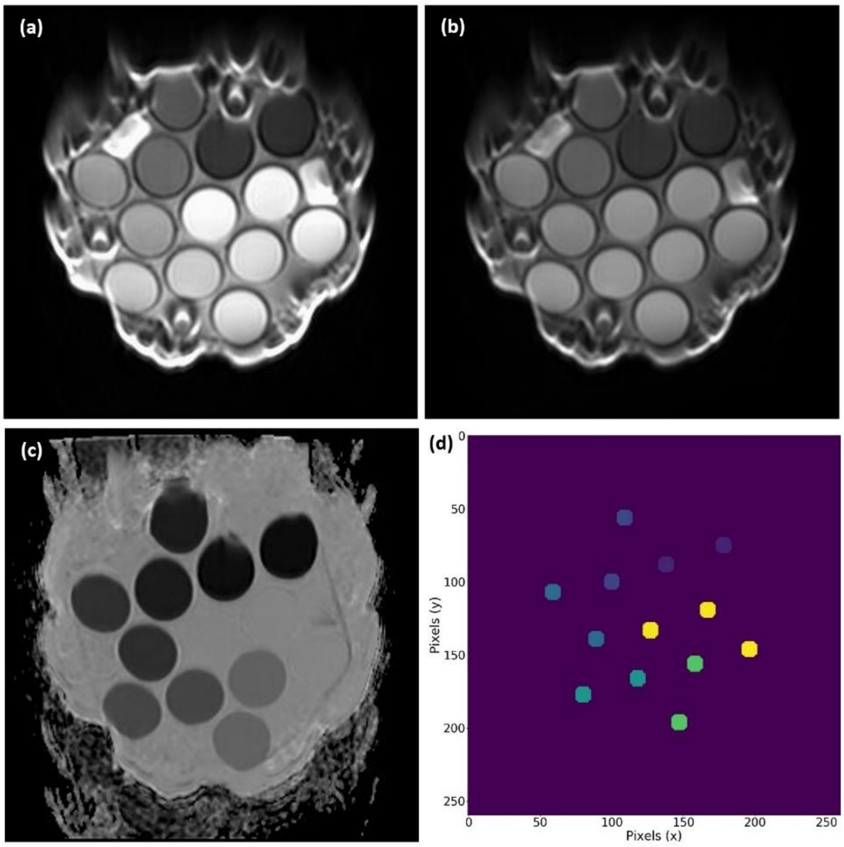

Supplementary Figure S-2: Month 7, repetition 1, axial images of the phantom's central slice, including the (a) isotropic trace diffusion weighted images with *b*-value = 0 s/mm^2 and (b) *b*-value = 500 s/mm^2. (c) Also, inline derived ADC-map with (d) corresponding ADC regions of interest for all 13 vials.
