# Supplementary material for: Conformance of a 3T radiotherapy MRI scanner to the QIBA Diffusion Profile
Source: Med Phys. 2022 Apr 11;49(7):4508–17. doi: 10.1002/mp.15645 (PMC9543906; doi:10.1002/mp.15645)
Supplement: Supplementary file 3 — Figure S3 [file MP-49-4508-s005.pdf]

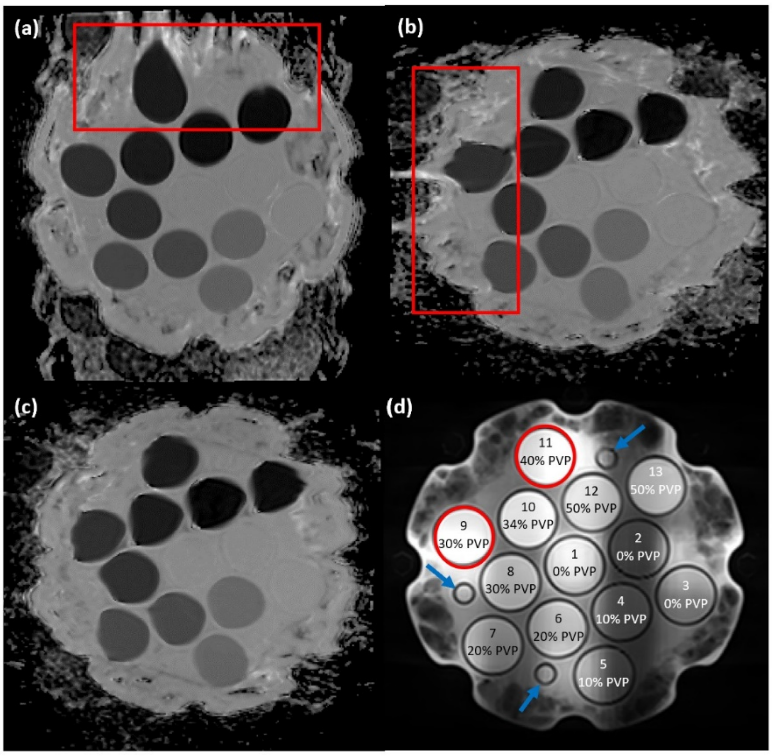

Supplementary Figure S-3: Month 7, repetition 1, central slices of the inline derived ADC maps for (a) axial, (b) sagittal and (c) coronal acquisitions. Both axial and sagittal acquisitions require the phantom to be rotated such that certain vials of the phantom (in locations indicated by the overlaid red boxes) are at the phantom's most anterior point. In theory, any air bubbles present in the ice-bath will migrate towards these locations due to gravity, making vials within at higher risk of being affected by susceptibility-induced distortions. Further, the three 5 mL fiducial markers, indicated by the blue arrows in the localiser image (d), increase the likelihood of bubbles being trapped closer to the higher concentrated PVP vials, for any directional acquisition. Vial numbers and PVP concentrations (%) have been included on the localiser to assist in visualisation, noting that vials most at risk of the susceptibility-induced distortions are circled in red.
