# Supplementary material for: Conformance of a 3T radiotherapy MRI scanner to the QIBA Diffusion Profile
Source: Med Phys. 2022 Apr 11;49(7):4508–17. doi: 10.1002/mp.15645 (PMC9543906; doi:10.1002/mp.15645)
Supplement: Supplementary file 4 — Figure S4 [file MP-49-4508-s006.pdf]

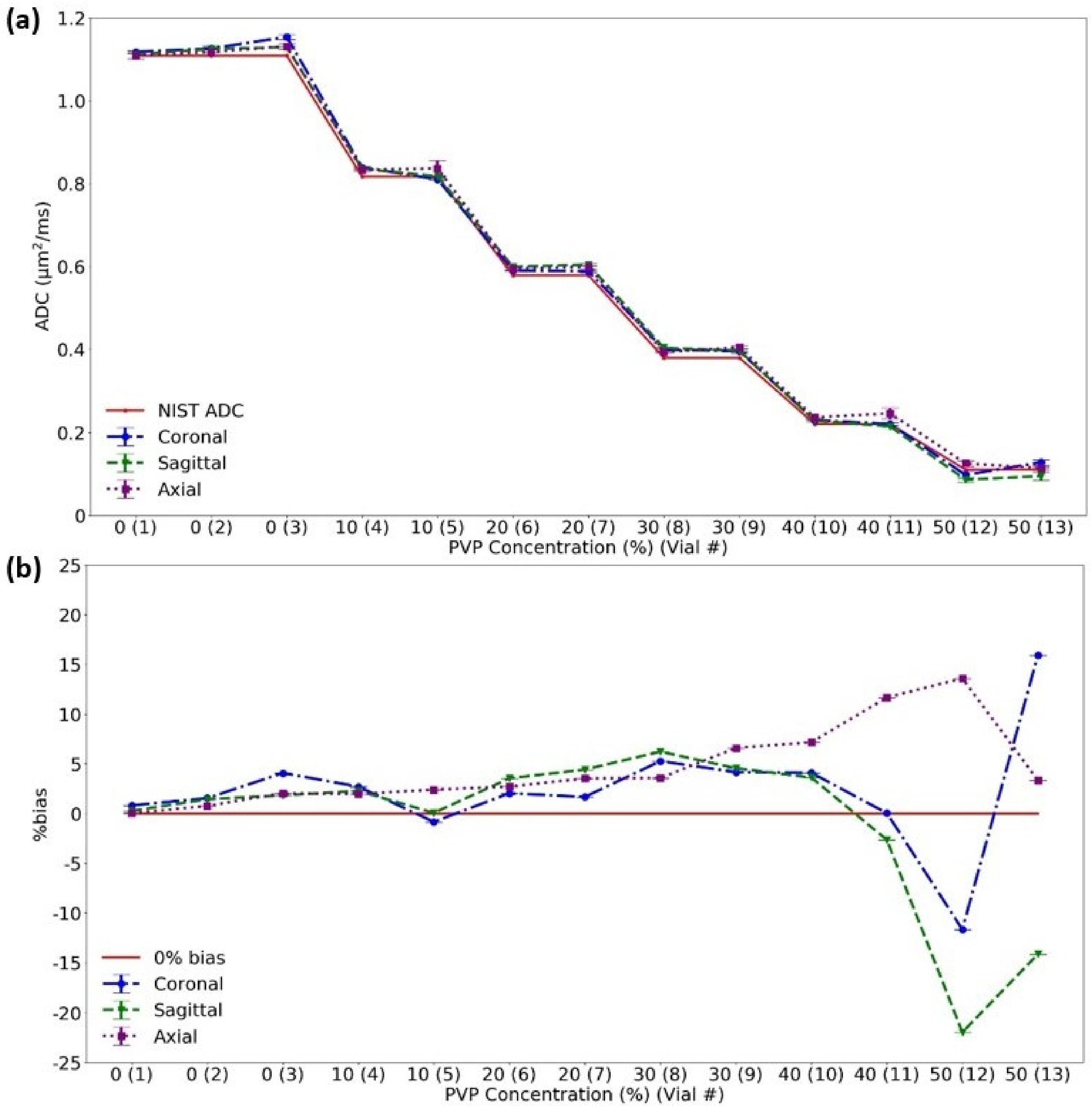

Supplementary Figure S-4: Graphical depiction of Supplementary Table S-1 data; (a) Presenting the mean and standard deviation (SD) of the Apparent Diffusion Coefficient (ADC) value (μm²/ms) measured over the 12 monthly acquisitions at 0 °C for each orthogonal imaging direction; and (b) Presenting the same values as a %difference from the NIST ADC value. Note that vial 1 represents the central water vial with 0% PVP concentration.
