# Supplementary material for: Conformance of a 3T radiotherapy MRI scanner to the QIBA Diffusion Profile
Source: Med Phys. 2022 Apr 11;49(7):4508–17. doi: 10.1002/mp.15645 (PMC9543906; doi:10.1002/mp.15645)
Supplement: Supplementary file 5 — Figure S5 [file MP-49-4508-s002.pdf]

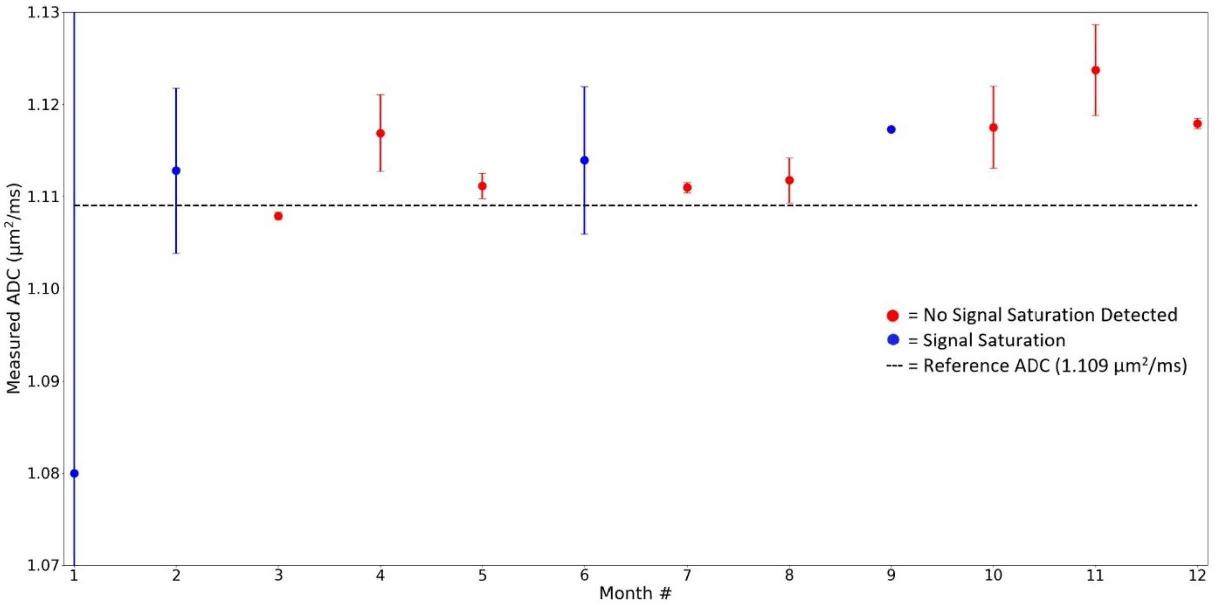

Supplementary Figure S-5: Observed monthly deviations for the central water vial ADC value over the 12-month study. Only the first repetition of axial acquisitions has been included, with ADC maps derived offline. The ADC is thus presented as the ADC value ± SD (calculated using mean square error). Note the SD for month 1 extended to ± 0.578 $\mu m^2/ms$.
