# Supplementary material for: Conformance of a 3T radiotherapy MRI scanner to the QIBA Diffusion Profile
Source: Med Phys. 2022 Apr 11;49(7):4508–17. doi: 10.1002/mp.15645 (PMC9543906; doi:10.1002/mp.15645)
Supplement: Supplementary file 6 — Figure S6 [file MP-49-4508-s007.pdf]

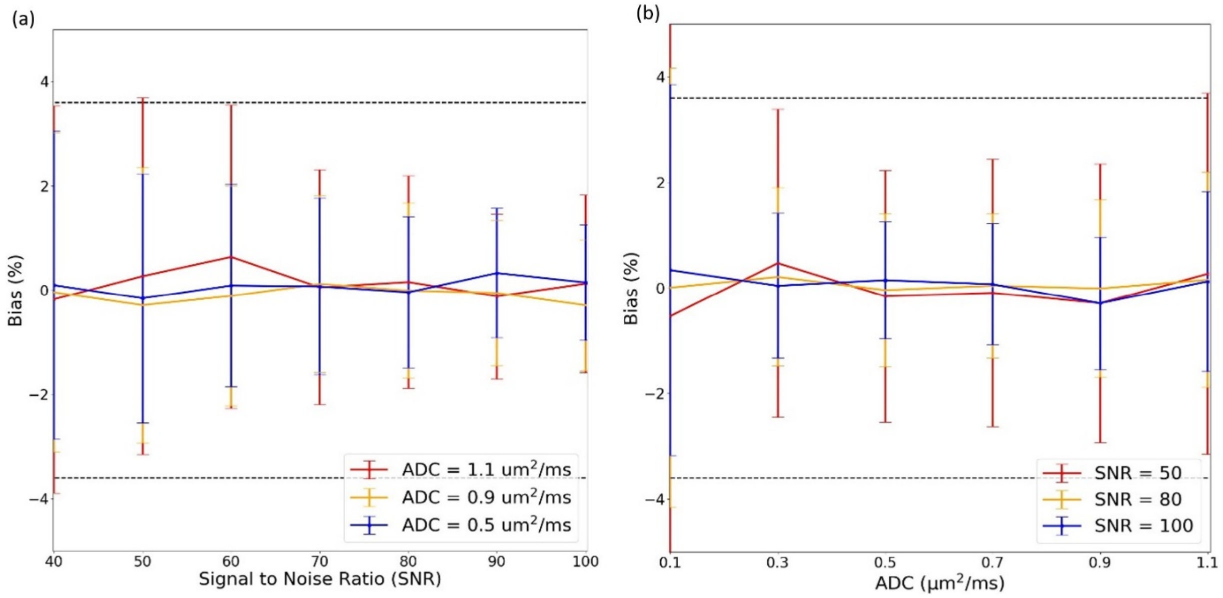

Supplementary Figure S-6: Examples of %bias for DWI-digital reference object (DRO) ADC maps derived offline. %bias and SD (error-bars) are presented with respect to input (a) ADC, and (b) signal-to-noise ratios (SNRs), over phantom relevant SNR and ADC DRO regions of interest, respectively. The Profile %bias tolerance of ± 3.6% has also been included.
