# Supplementary material for: Conformance of a 3T radiotherapy MRI scanner to the QIBA Diffusion Profile
Source: Med Phys. 2022 Apr 11;49(7):4508–17. doi: 10.1002/mp.15645 (PMC9543906; doi:10.1002/mp.15645)
Supplement: Supplementary file 7 — Table S1 [file MP-49-4508-s001.doc]

Supplementary Table S-1: Mean and standard deviation (SD) of the Apparent Diffusion Coefficient (ADC) value (µm^2^/ms) measured over the 12 monthly acquisitions at 0 ^o^C, and for each orthogonal imaging direction. **^a^**National Institute of Standards and Technology (NIST) reference ADC values have also been presented: measured for the same batch of Polyvinylpyrrolidone (PVP) samples as those embedded in the phantom’s vials, at 0 ^o^C.

| Vial #: | | | 1 | 2 | 3 | 4 | 5 | 6 | 7 | 8 | 9 | 10 | 11 | 12 | 13 |
| --- | --- | --- | --- | --- | --- | --- | --- | --- | --- | --- | --- | --- | --- | --- | --- |
| **ADC** | PVP (%): | | 0 | 0 | 0 | 10 | 10 | 20 | 20 | 30 | 30 | 40 | 40 | 50 | 50 |
| **NIST^a^** | | Value | 1.109 | 1.109 | 1.109 | 0.817 | 0.817 | 0.579 | 0.579 | 0.380 | 0.380 | 0.220 | 0.220 | 0.110 | 0.110 |
|  |  | SD | 0.025 | 0.025 | 0.025 | 0.019 | 0.019 | 0.015 | 0.015 | 0.011 | 0.011 | 0.007 | 0.007 | 0.005 | 0.005 |
| **Coronal** | | Value | 1.118 | 1.126 | 1.154 | 0.839 | 0.810 | 0.591 | 0.589 | 0.400 | 0.396 | 0.229 | 0.220 | 0.097 | 0.128 |
|  |  | SD | 0.004 | 0.004 | 0.006 | 0.002 | 0.002 | 0.001 | 0.003 | 0.003 | 0.005 | 0.002 | 0.003 | 0.009 | 0.007 |
| **Sagittal** | | Value | 1.112 | 1.125 | 1.129 | 0.836 | 0.818 | 0.600 | 0.605 | 0.404 | 0.397 | 0.228 | 0.214 | 0.086 | 0.094 |
|  |  | SD | 0.010 | 0.008 | 0.003 | 0.002 | 0.003 | 0.001 | 0.003 | 0.003 | 0.004 | 0.003 | 0.003 | 0.007 | 0.010 |
| **Axial** | | Value | 1.110 | 1.118 | 1.132 | 0.834 | 0.837 | 0.595 | 0.600 | 0.394 | 0.406 | 0.236 | 0.245 | 0.125 | 0.114 |
|  |  | SD | 0.010 | 0.009 | 0.007 | 0.004 | 0.020 | 0.004 | 0.003 | 0.002 | 0.003 | 0.003 | 0.013 | 0.006 | 0.003 |
